# Complete Genome Sequence of *Mycoplasma californicum* Strain HAZ160_1 from Bovine Mastitic Milk in Japan

**DOI:** 10.1128/genomeA.00684-14

**Published:** 2014-07-10

**Authors:** Eiji Hata, Kenji Murakami

**Affiliations:** a Dairy Hygiene Research Division, National Institute of Animal Health (NIAH), National Agriculture and Food Research Organization (NARO), Sapporo, Hokkaido, Japan; b Department of Veterinary Medicine, Faculty of Agriculture, Iwate University, Morioka, Iwate, Japan

## Abstract

Bovine mycoplasmal mastitis is spreading quickly among cows. It often leads to clinical mastitis outbreaks and often results in huge economic losses. *Mycoplasma californicum* is an important causal species of bovine mastitis. Presented here is the 799,088-bp complete genome sequence of *M. californicum* strain HAZ160_1, which was isolated in Japan.

## GENOME ANNOUNCEMENT

*Mycoplasma californicum* is a causal bacterium of bovine mastitis, arthritis, and pneumonia as well as an indigenous bacterium affecting cattle (1, 2). Mastitis due to *M. californicum* features strong infectivity, severe symptoms, and poor response to treatment with antibiotics and is often accompanied by major economic losses (2–4). Despite its importance, little genetic information on *M. californicum* is available. Presented here is the whole-genome sequence of strain HAZ 160_1, which was isolated in 2008 from bovine mastitic milk in Japan.

Total genomic DNA was prepared from *M. californicum* strain HAZ 160_1 and subjected to 454 Titanium sequencing at the Hokkaido System Science Co., Ltd., Sapporo, Japan. The resulting reads were assembled *de novo* using GS *de novo* Assembler software version 2.7 (Roche), yielding 36 contigs with 94.8× coverage. An analysis of the contig ends together with PCR amplification and amplicon cloning showed that the 799,088-bp genome had a closed-ring structure. After the initial automated annotation performed using the Microbial Genome Annotation Pipeline version 2.18 at the DNA Data Bank of Japan (http://migap.ddbj.nig.ac.jp/mgap/jsp/index.jsp) (5–7), manual curation was performed, followed by verification of potential pseudogenes by PCR and Sanger sequencing. As a result, we confirmed 574 open reading frames, 15 pseudogenes, 31 tRNAs, and 2 sets of each rRNA (5S rRNA, 16S rRNA, and 23S rRNA) in this genome sequence. Moreover, the G+C content was 30.8%.

As anticipated based on its 16S rRNA-based phylogeny, most genes in *M. californicum* strain HAZ 160_1 exhibited high similarity to the amino acid sequences of the genes encoding members of the *Mycoplasma fermentans* cluster, with the greatest similarity shown with genes from the bovine pathogen *Mycoplasma bovigenitalium* (8).

The hypothetical proteins MCAL160_0738, MCAL160_0902, MCAL160_0908, and MCAL160_0912 may be involved in the antigenic variation shift in surface proteins that plays a role in the adaptation to new surroundings and in host defense mechanisms (9). A part of the amino acid sequences of these genes showed certain similarity to the membrane proteins of other *Mycoplasma* and *Ureaplasma* species. Moreover, the discriminative homopolymeric tract of contiguous thymines (Poly-T) is located upstream of repetitive regions in the hypothetical proteins MCAL160_0738, MCAL160_0908, and MCAL160_0912 (9). These genes contain repetitive regions encoding distinctive periodic amino acid sequences, and the number of repetitive units differed among *M. californicum* strains.

Although this genome sequence contained genes of proteins involved in the synthesis of capsular polysaccharides, which are suggested to be important mycoplasmal etiologic agents, i.e., UTP-glucose-1-phosphate-uridyltransferase and glycosyltransferases, etc., the genes of proteins involved in the production of active oxygen-containing molecules, which are also suggested to be important etiologic agents, were not confirmed (10).

The genomic sequence of *M. californicum* will provide a foundation for the investigation of this species in the future. Ultimately, it is hoped that this study will contribute to the reduction of bovine diseases such as mastitis or pneumonia.

### Nucleotide sequence accession number.

This whole-genome sequence has been registered at DDBJ/EMBL/GenBank under accession no. AP013353.
